# Supplementary material for: Inadequate preparedness for response to COVID-19 is associated with stress and burnout among healthcare workers in Ghana
Source: PLoS One. 2021 Apr 16;16(4):e0250294. doi: 10.1371/journal.pone.0250294 (PMC8051822; doi:10.1371/journal.pone.0250294)
Supplement: S1 Appendix — (DOCX) [file pone.0250294.s001.docx]

| **S1 Appendix: Perceived stress items** | | |
| --- | --- | --- |
|  |  |  |
| 1. In the past month, how often have you been upset because of something that happened unexpectedly? | | |
| 0, Never |  |  |
| 1, Almost Never |  |  |
| 2, Sometimes |  |  |
| 3, Fairly Often |  |  |
| 4, Very Often |  |  |
|  |  |  |
| 2. In the past month, how often have you felt unable to control the important things in your life? | | |
| 0, Never |  |  |
| 1, Almost Never |  |  |
| 2, Sometimes |  |  |
| 3, Fairly Often |  |  |
| 4, Very Often |  |  |
|  |  |  |
| 3. In the past month, how often have you felt nervous or stressed? | | |
| 0, Never |  |  |
| 1, Almost Never |  |  |
| 2, Sometimes |  |  |
| 3, Fairly Often |  |  |
| 4, Very Often |  |  |
|  |  |  |
| 4. In the past month, how often have you felt confident about your ability to handle personal problems? | | |
| 0, Never |  |  |
| 1, Almost Never |  |  |
| 2, Sometimes |  |  |
| 3, Fairly Often |  |  |
| 4, Very Often |  |  |
|  |  |  |
| 5. In the past month, how often have you felt that things were going your way? | | |
| 0, Never |  |  |
| 1, Almost Never |  |  |
| 2, Sometimes |  |  |
| 3, Fairly Often |  |  |
| 4, Very Often |  |  |
|  |  |  |
| 6. In the past month, how often have you found that you could not cope with all the things you had to do? | | |
| 0, Never |  |  |
| 1, Almost Never |  |  |
| 2, Sometimes |  |  |
| 3, Fairly Often |  |  |
| 4, Very Often |  |  |
|  |  |  |
| 7. In the past month, how often have you been able to control irritations in your life? | | |
| 0, Never |  |  |
| 1, Almost Never |  |  |
| 2, Sometimes |  |  |
| 3, Fairly Often |  |  |
| 4, Very Often |  |  |
|  |  |  |
| 8. In the past month, how often have you felt that you were on top of things? | | |
| 0, Never |  |  |
| 1, Almost Never |  |  |
| 2, Sometimes |  |  |
| 3, Fairly Often |  |  |
| 4, Very Often |  |  |
|  |  |  |
| 9. In the past month, how often have you been angry because of things that happened that were outside of your control? | | |
| 0, Never |  |  |
| 1, Almost Never |  |  |
| 2, Sometimes |  |  |
| 3, Fairly Often |  |  |
| 4, Very Often |  |  |
|  |  |  |
| 10. In the past month, how often have you felt that difficulties were piling up so high that you could not overcome them? | | |
| 0, Never |  |  |
| 1, Almost Never |  |  |
| 2, Sometimes |  |  |
| 3, Fairly Often |  |  |
| 4, Very Often |  |  |
