# Supplementary material for: Inadequate preparedness for response to COVID-19 is associated with stress and burnout among healthcare workers in Ghana
Source: PLoS One. 2021 Apr 16;16(4):e0250294. doi: 10.1371/journal.pone.0250294 (PMC8051822; doi:10.1371/journal.pone.0250294)
Supplement: S2 Appendix — (DOCX) [file pone.0250294.s002.docx]

|  | **S2 Appendix: Burnout items** | | |
| --- | --- | --- | --- |
| Domain | Item |  |  |
| ***Physical Fatigue*** |  |  |  |
|  | 1. I feel tired |  |  |
|  | 1, Never or almost never |  |  |
|  | 2, Very infrequently |  |  |
|  | 3, Quite infrequently |  |  |
|  | 4, Sometimes |  |  |
|  | 5, Quite frequently |  |  |
|  | 6, Very frequently |  |  |
|  | 7, Always or almost always |  |  |
|  |  |  |  |
|  | 2. I have no energy for going to work in the morning | |  |
|  | 1 Never or almost never |  |  |
|  | 2 Very infrequently |  |  |
|  | 3 Quite infrequently |  |  |
|  | 4 Sometimes |  |  |
|  | 5 Quite frequently |  |  |
|  | 6 Very frequently |  |  |
|  | 7 Always or almost always |  |  |
|  |  |  |  |
|  | 3. I feel physically drained |  |  |
|  | 1 Never or almost never |  |  |
|  | 2 Very infrequently |  |  |
|  | 3 Quite infrequently |  |  |
|  | 4 Sometimes |  |  |
|  | 5 Quite frequently |  |  |
|  | 6 Very frequently |  |  |
|  | 7 Always or almost always |  |  |
|  |  |  |  |
|  | 4. I feel fed up |  |  |
|  | 1 Never or almost never |  |  |
|  | 2 Very infrequently |  |  |
|  | 3 Quite infrequently |  |  |
|  | 4 Sometimes |  |  |
|  | 5 Quite frequently |  |  |
|  | 6 Very frequently |  |  |
|  | 7 Always or almost always |  |  |
|  |  |  |  |
|  | 5. I feel like my "batteries" are "dead" |  |  |
|  | 1 Never or almost never |  |  |
|  | 2 Very infrequently |  |  |
|  | 3 Quite infrequently |  |  |
|  | 4 Sometimes |  |  |
|  | 5 Quite frequently |  |  |
|  | 6 Very frequently |  |  |
|  | 7 Always or almost always |  |  |
|  |  |  |  |
|  | 6. I feel burned out |  |  |
|  | 1 Never or almost never |  |  |
|  | 2 Very infrequently |  |  |
|  | 3 Quite infrequently |  |  |
|  | 4 Sometimes |  |  |
|  | 5 Quite frequently |  |  |
|  | 6 Very frequently |  |  |
|  | 7 Always or almost always |  |  |
|  |  |  |  |
| ***Cognitive weariness*** |  |  |  |
|  | 7. My thinking process is slow |  |  |
|  | 1 Never or almost never |  |  |
|  | 2 Very infrequently |  |  |
|  | 3 Quite infrequently |  |  |
|  | 4 Sometimes |  |  |
|  | 5 Quite frequently |  |  |
|  | 6 Very frequently |  |  |
|  | 7 Always or almost always |  |  |
|  |  |  |  |
|  | 8. I have difficulty concentrating |  |  |
|  | 1 Never or almost never |  |  |
|  | 2 Very infrequently |  |  |
|  | 3 Quite infrequently |  |  |
|  | 4 Sometimes |  |  |
|  | 5 Quite frequently |  |  |
|  | 6 Very frequently |  |  |
|  | 7 Always or almost always |  |  |
|  |  |  |  |
|  | 9. I feel I'm not thinking clearly |  |  |
|  | 1 Never or almost never |  |  |
|  | 2 Very infrequently |  |  |
|  | 3 Quite infrequently |  |  |
|  | 4 Sometimes |  |  |
|  | 5 Quite frequently |  |  |
|  | 6 Very frequently |  |  |
|  | 7 Always or almost always |  |  |
|  |  |  |  |
|  | 10. I feel I'm not focused in my thinking |  |  |
|  | 1 Never or almost never |  |  |
|  | 2 Very infrequently |  |  |
|  | 3 Quite infrequently |  |  |
|  | 4 Sometimes |  |  |
|  | 5 Quite frequently |  |  |
|  | 6 Very frequently |  |  |
|  | 7 Always or almost always |  |  |
|  |  |  |  |
|  | 11. I have difficulty thinking about complex things | |  |
|  | 1 Never or almost never |  |  |
|  | 2 Very infrequently |  |  |
|  | 3 Quite infrequently |  |  |
|  | 4 Sometimes |  |  |
|  | 5 Quite frequently |  |  |
|  | 6 Very frequently |  |  |
|  | 7 Always or almost always |  |  |
|  |  |  |  |
| ***Emotional exhaustion*** | |  |  |
|  | 12. I feel I am unable to be sensitive to the needs of coworkers and patients | | |
|  | 1 Never or almost never |  |  |
|  | 2 Very infrequently |  |  |
|  | 3 Quite infrequently |  |  |
|  | 4 Sometimes |  |  |
|  | 5 Quite frequently |  |  |
|  | 6 Very frequently |  |  |
|  | 7 Always or almost always |  |  |
|  |  |  |  |
|  | 13. I feel I am not capable of investing emotionally in coworkers and patients | | |
|  | 1 Never or almost never |  |  |
|  | 2 Very infrequently |  |  |
|  | 3 Quite infrequently |  |  |
|  | 4 Sometimes |  |  |
|  | 5 Quite frequently |  |  |
|  | 6 Very frequently |  |  |
|  | 7 Always or almost always |  |  |
|  |  |  |  |
|  | 14. I feel I am not capable of being sympathetic to co-workers and patients | | |
|  | 1 Never or almost never |  |  |
|  | 2 Very infrequently |  |  |
|  | 3 Quite infrequently |  |  |
|  | 4 Sometimes |  |  |
|  | 5 Quite frequently |  |  |
|  | 6 Very frequently |  |  |
|  | 7 Always or almost always |  |  |
