# Supplementary material for: Inadequate preparedness for response to COVID-19 is associated with stress and burnout among healthcare workers in Ghana
Source: PLoS One. 2021 Apr 16;16(4):e0250294. doi: 10.1371/journal.pone.0250294 (PMC8051822; doi:10.1371/journal.pone.0250294)
Supplement: S3 Appendix — (DOCX) [file pone.0250294.s003.docx]

| **S3 Appendix: Perceived preparedness items** | | |
| --- | --- | --- |
|  |  |  |
| **Appendix 1: Distribution of Responses to individual preparedness questions** | | |
|  |  |  |
| 1. How prepared are you as a health care provider in your ability to assess and triage patients with acute respiratory symptoms? | | |
| 0, Not at all prepared |  |  |
| 1, A little prepared |  |  |
| 2, Prepared |  |  |
| 3, Very prepared |  |  |
| 5, Not applicable to my role | |  |
|  |  |  |
| 2. How prepared are you as a health care provider in your ability to diagnose a patient with COVID-19? | | |
| 0, Not at all prepared |  |  |
| 1, A little prepared |  |  |
| 2, Prepared |  |  |
| 3, Very prepared |  |  |
| 4, I don't know about this | |  |
| 5, Not applicable to my role | |  |
|  |  |  |
| 3. How prepared are you as a health care provider in your ability to manage a patient with diagnosed COVID-19? | | |
| 0, Not at all prepared |  |  |
| 1, A little prepared |  |  |
| 2, Prepared |  |  |
| 3, Very prepared |  |  |
| 4, I don't know about this | |  |
| 5, Not applicable to my role | |  |
|  |  |  |
| 4. How prepared are you as a health care provider in your ability to accurately put on PPE? | | |
| 0, Not at all prepared |  |  |
| 1, A little prepared |  |  |
| 2, Prepared |  |  |
| 3, Very prepared |  |  |
| 4, I don't know about this | |  |
|  |  |  |
| 5. How prepared are you as a health care provider in your ability to safely take take off PPE? | | |
| 0, Not at all prepared |  |  |
| 1, A little prepared |  |  |
| 2, Prepared |  |  |
| 3, Very prepared |  |  |
| 4, I don't know about this | |  |
|  |  |  |
| 6. How prepared are you as a health care provider in your ability to implement standard contact precautions? | | |
| 0, Not at all prepared |  |  |
| 1, A little prepared |  |  |
| 2, Prepared |  |  |
| 3, Very prepared |  |  |
| 4, I don't know about this | |  |
|  |  |  |
|  |  |  |
| 7. How prepared are you as a health care provider in your ability to implement standard airborne precautions? | | |
| 0, Not at all prepared |  |  |
| 1, A little prepared |  |  |
| 2, Prepared |  |  |
| 3, Very prepared |  |  |
| 4, I don't know about this | |  |
|  |  |  |
| 8. How prepared are you as a health care provider in your ability to communicate COVID-19 risks to your patients? | | |
| 0, Not at all prepared |  |  |
| 1, A little prepared |  |  |
| 2, Prepared |  |  |
| 3, Very prepared |  |  |
|  |  |  |
| 9. How prepared are you as a health care provider in your ability to educate the public about COVID-19? | | |
| 0, Not at all prepared |  |  |
| 1, A little prepared |  |  |
| 2, Prepared |  |  |
| 3, Very prepared |  |  |
|  |  |  |
| 10. How prepared are you as a health care provider in your ability to ration scarce life-saving commodities? | | |
| 0, Not at all prepared |  |  |
| 1, A little prepared |  |  |
| 2, Prepared |  |  |
| 3, Very prepared |  |  |
| 4, I don't know about this | |  |
|  |  |  |
| 11. As a health care provider, how mentally prepared are you to attend to a person diagnosed with COVID-19? | | |
| 0, Not at all prepared |  |  |
| 1, A little prepared |  |  |
| 2, Prepared |  |  |
| 3, Very prepared |  |  |
| 4, I don't know about this | |  |
|  |  |  |
| 12. How prepared is the health facility you work in to diagnose COVID-19? | | |
| 0, Not at all prepared |  |  |
| 1, A little prepared |  |  |
| 2, Prepared |  |  |
| 3, Very prepared |  |  |
| 4, I don't know about this | |  |
|  |  |  |
| 13. How prepared is the health facility you work in to manage patients diagnosed with COVID-19? | | |
| 0, Not at all prepared |  |  |
| 1, A little prepared |  |  |
| 2, Prepared |  |  |
| 3, Very prepared |  |  |
| 4, I don't know about this | |  |
|  |  |  |
| 14. How prepared is the health facility you work in to prevent the spread of COVID-19 to other patients and health workers if you had an infected patient? | | |
| 0, Not at all prepared |  |  |
| 1, A little prepared |  |  |
| 2, Prepared |  |  |
| 3, Very prepared |  |  |
| 4, I don't know about this | |  |
|  |  |  |
| 15. How prepared is the health facility you work in to prevent spread of COVID-19 to other patients and health workers if you had an infected health worker? | | |
| 0, Not at all prepared |  |  |
| 1, A little prepared |  |  |
| 2, Prepared |  |  |
| 3, Very prepared |  |  |
| 4, I don't know about this | |  |
