# Supplementary material for: Inadequate preparedness for response to COVID-19 is associated with stress and burnout among healthcare workers in Ghana
Source: PLoS One. 2021 Apr 16;16(4):e0250294. doi: 10.1371/journal.pone.0250294 (PMC8051822; doi:10.1371/journal.pone.0250294)
Supplement: S4 Appendix — (DOCX) [file pone.0250294.s004.docx]

| **S4 Appendix: Perceived knowledge items** |
| --- |
| 1. Do you know what to do if you suspect a patient may have COVID-19? |
| 0, No |
| 1, Somewhat |
| 2, Yes |
|  |
| 2. Do you know how to manage a confirmed case of COVID-19? |
| 0, No |
| 1, Somewhat |
| 2, Yes |
| 3, Not applicable to my role |
|  |
|  |
|  |
|  |
|  |
|  |
