# Supplementary material for: Inadequate preparedness for response to COVID-19 is associated with stress and burnout among healthcare workers in Ghana
Source: PLoS One. 2021 Apr 16;16(4):e0250294. doi: 10.1371/journal.pone.0250294 (PMC8051822; doi:10.1371/journal.pone.0250294)
Supplement: S6 Appendix — (DOCX) [file pone.0250294.s006.docx]

| **S6 Appendix: Comparing Sample excluded to analytic samples** | | | | | | | | | | | | | | | | |
| --- | --- | --- | --- | --- | --- | --- | --- | --- | --- | --- | --- | --- | --- | --- | --- | --- |
|  | **Total** | |  | **Perceived stress analysis** | | | | | |  | **Burnout analysis** | | | | |  |
| **Variables** |  |  |  | **Excluded** | |  | **Included** | |  |  | **Excluded** | |  | **Included** | |  |
|  | **No.** | **%** |  | **No.** | **%** |  | **No.** | **%** | **p-value** |  | **No.** | **%** |  | **No.** | **%** | **p-value** |
| Position |  |  |  |  |  |  |  |  | 0.235 |  |  |  |  |  |  | 0.244 |
| Doctor | 116 | 18 |  | 34 | 14.7 |  | 82 | 19.8 |  |  | 35 | 14.8 |  | 81 | 19.8 |  |
| Nurse/related | 410 | 63.5 |  | 151 | 65.1 |  | 259 | 62.6 |  |  | 154 | 65 |  | 256 | 62.6 |  |
| Other | 120 | 18.6 |  | 47 | 20.3 |  | 73 | 17.6 |  |  | 48 | 20.3 |  | 72 | 17.6 |  |
| Total | 646 | 100 |  | 232 | 100 |  | 414 | 100 |  |  | 237 | 100 |  | 409 | 100 |  |
| Region |  |  |  |  |  |  |  |  | 0.883 |  |  |  |  |  |  | 0.91 |
| Greater Accra/Ashanti | 150 | 23.5 |  | 56 | 25 |  | 94 | 22.7 |  |  | 57 | 24.9 |  | 93 | 22.7 |  |
| Northern region | 142 | 22.3 |  | 48 | 21.4 |  | 94 | 22.7 |  |  | 50 | 21.8 |  | 92 | 22.5 |  |
| Other Northern | 144 | 22.6 |  | 48 | 21.4 |  | 96 | 23.2 |  |  | 49 | 21.4 |  | 95 | 23.2 |  |
| other Southern | 202 | 31.7 |  | 72 | 32.1 |  | 130 | 31.4 |  |  | 73 | 31.9 |  | 129 | 31.5 |  |
| Total | 638 | 100 |  | 224 | 100 |  | 414 | 100 |  |  | 229 | 100 |  | 409 | 100 |  |
| Facility type |  |  |  |  |  |  |  |  | 0.027 |  |  |  |  |  |  |  |
| Teaching hospital | 149 | 23.2 |  | 40 | 17.5 |  | 109 | 26.3 |  |  | 41 | 17.5 |  | 108 | 26.4 |  |
| Other government facility | 401 | 62.4 |  | 157 | 68.6 |  | 244 | 58.9 |  |  | 160 | 68.4 |  | 241 | 58.9 |  |
| Private/mission facility | 93 | 14.5 |  | 32 | 14 |  | 61 | 14.7 |  |  | 33 | 14.1 |  | 60 | 14.7 |  |
| Total | 643 | 100 |  | 229 | 100 |  | 414 | 100 |  |  | 234 | 100 |  | 409 | 100 |  |
| Years of experience |  |  |  |  |  |  |  |  | 0.28 |  |  |  |  |  |  | 0.375 |
| 5 or less years | 220 | 34.5 |  | 86 | 38.4 |  | 134 | 32.4 |  |  | 87 | 38 |  | 133 | 32.5 |  |
| 6 to 10 years | 255 | 40 |  | 82 | 36.6 |  | 173 | 41.8 |  |  | 86 | 37.6 |  | 169 | 41.3 |  |
| More than 10 years | 163 | 25.5 |  | 56 | 25 |  | 107 | 25.8 |  |  | 56 | 24.5 |  | 107 | 26.2 |  |
| Total | 638 | 100 |  | 224 | 100 |  | 414 | 100 |  |  | 229 | 100 |  | 409 | 100 |  |
| Age |  |  |  |  |  |  |  |  | 0.121 |  |  |  |  |  |  | 0.098 |
| Less than 30 | 190 | 30 |  | 77 | 34.5 |  | 113 | 27.5 |  |  | 79 | 34.6 |  | 111 | 27.3 |  |
| 30 to 39 | 343 | 54.1 |  | 109 | 48.9 |  | 234 | 56.9 |  |  | 111 | 48.7 |  | 232 | 57.1 |  |
| 40 to 73 | 101 | 15.9 |  | 37 | 16.6 |  | 64 | 15.6 |  |  | 38 | 16.7 |  | 63 | 15.5 |  |
| Total | 634 | 100 |  | 223 | 100 |  | 411 | 100 |  |  | 228 | 100 |  | 406 | 100 |  |
| Gender |  |  |  |  |  |  |  |  | 0.119 |  |  |  |  |  |  | 0.105 |
| Male | 311 | 48.4 |  | 101 | 44.3 |  | 210 | 50.7 |  |  | 103 | 44.2 |  | 208 | 50.9 |  |
| Female | 331 | 51.6 |  | 127 | 55.7 |  | 204 | 49.3 |  |  | 130 | 55.8 |  | 201 | 49.1 |  |
| Total | 642 | 100 |  | 228 | 100 |  | 414 | 100 |  |  | 233 | 100 |  | 409 | 100 |  |
| Parity |  |  |  |  |  |  |  |  | 0.209 |  |  |  |  |  |  | 0.104 |
| No children | 202 | 32.6 |  | 78 | 36.3 |  | 124 | 30.6 |  |  | 81 | 36.8 |  | 121 | 30.2 |  |
| 1 or 2 children | 274 | 44.2 |  | 85 | 39.5 |  | 189 | 46.7 |  |  | 85 | 38.6 |  | 189 | 47.2 |  |
| 3 to 6 children | 144 | 23.2 |  | 52 | 24.2 |  | 92 | 22.7 |  |  | 54 | 24.5 |  | 90 | 22.5 |  |
| Total | 620 | 100 |  | 215 | 100 |  | 405 | 100 |  |  | 220 | 100 |  | 400 | 100 |  |
| Marital status |  |  |  |  |  |  |  |  | 0.044 |  |  |  |  |  |  | 0.058 |
| Single | 203 | 31.7 |  | 83 | 36.7 |  | 120 | 29 |  |  | 84 | 36.4 |  | 119 | 29.1 |  |
| Married | 437 | 68.3 |  | 143 | 63.3 |  | 294 | 71 |  |  | 147 | 63.6 |  | 290 | 70.9 |  |
| Total | 640 | 100 |  | 226 | 100 |  | 414 | 100 |  |  | 231 | 100 |  | 409 | 100 |  |
