# Supplementary material for: Inadequate preparedness for response to COVID-19 is associated with stress and burnout among healthcare workers in Ghana
Source: PLoS One. 2021 Apr 16;16(4):e0250294. doi: 10.1371/journal.pone.0250294 (PMC8051822; doi:10.1371/journal.pone.0250294)
Supplement: Attachment — Submitted filename: Response to reviewers.docx [file pone.0250294.s007.docx]

Dear Dr. Lionetti

Thank you for the opportunity to revise and resubmit our manuscript. We have given careful consideration to all the issues raised by the reviewers and revised our manuscript accordingly. Below is a point-by-point clarification and explanation of revisions made in response to the reviewers.

Sincerely,

Manuscript authors

| ***Comments*** | ***Response*** |
| --- | --- |
| Journal comments | |
| Please ensure that your manuscript meets PLOS ONE's style requirements, including those for file naming. | We have revised the manuscript to meet PLOS ONE’s style requirements and name files according to the journal’s guidelines. We have also added the following sections: “contributors”, “data availability”, “Funding”, and competing interests after the conclusions section, on page 20. |
| Please include captions for your Supporting Information files at the end of your manuscript, and update any in-text citations to match accordingly. | We have renamed our Supporting Information files to reflect the journal’s guidelines, added a Supporting Information section after the references, on page 26, and revised in-text citations of the supporting information accordingly. |
| Reviewer #1: | |
| Covid-19 is the most salient topic of the year, from a health, social, and scientific point of view. The authors detailed the psychological (and psychopathological) impact of the pandemic on the most affected category in terms of mental well-being.  The statistical analysis carried out appears meticulous and well explained. The number of variables investigated is large and overall sufficient to realistically describe the distress condition of the HCWs. Through the survey, the authors characterize study participants with demographic, professional, cognitive, and emotional specifications. The constructs of stress and burnout, assessed by validated scales, undoubtedly reflect the difficulties faced by the HCWs, and the association with the insufficient perceived preparedness stimulates reflection about potential and necessary improvements in Ghana – and global – health system. | Thank you |
| Main concerns: |  |
| I suggest adding or clarifying some information about sampling and data selection methods.  1.Since respondents have the option to skip some questions, you should specify if and how many subjects were excluded from the analysis (i.e. has a cut-off been set? Have the stress and burnout questionnaires been included only if complete?) | Yes, we only used data from respondents who completed all of the stress and burnout questions. 646 people started the survey (i.e answered the first question), but many did not complete the survey. For the present study, we excluded data from 232 and 237 people from the stress and burnout analytic samples.  Many of the respondents we excluded answered only the first few questions in the survey and the stress and burnout questions were among the final set of questions. Thus, given that stress and burnout were the key outcomes in this paper, we did not think it was appropriate to impute for the missing data. We however compared the characteristics of those excluded (since the demographic questions were the initial questions and many just answered those) to those included.  We have added Appendix 3 to our revised manuscript, which shows that the analytic sample and excluded people were not substantially different. We have described this in the methods section on page 10 and in the sensitivity analysis on page 15. |
| 2.It is unclear whether the two samples (n = 414 and n = 409 for stress and burnout, respectively) overlap or if they are distinct groups (i.e did each subject answer to both questionnaires or only one of them, either burnout or stress?) | These two samples overlap. The difference is due to a few more missing observations on the burnout items. We have clarified this in the methods section on page 10. |
| In the last line of paragraph 2.2. you wrote that "Additional study methods can be found elsewhere (41)", but in the cited article I found an identical description. In this regard, given the mention and the similarities, it would be appropriate to elucidate the possible relationship between the two manuscripts. | The first study manuscript we referenced examines factors associated with perceived preparedness of healthcare workers. We referenced it for its description of the psychometric analyses used to validate the perceived preparedness scale. In our submitted manuscript on burnout and stress, perceived preparedness is a key predictor, however we did not go into details in our methods section on the psychometric analyses/validation methods. Thus, we referenced the first manuscript for those methods. We have clarified this in the revised manuscript. |
| Minor comments: |  |
| I noticed that several times you mentioned online news sites (e.g. Reuters, GhanaWeb, CNN, Deutsche Well, ScienceDaily). I recommend, if and when possible, replacing them with references from scientific journals. | We have searched for articles in scientific journals for these references, however, we unfortunately did not find any for the Reuters, GhanaWeb, and CNN references, all of which discuss Ghanaian healthcare worker strike threats, cases, and related deaths from COVID-19. Unfortunately, these developments were reported in news articles and not in scientific reports.  We have however replaced the Deutsche Welle reference with a scientific journal article (Ref # 27) and removed the Science Daily reference since it discusses a scientific study that is already referenced in our manuscript (Ref # 50). |
| I found some typing errors in the list of references (ref. N ° 17, n ° 35). I recommend checking it out. | Thank you for flagging these. We have corrected the typing errors. |
|  | Thank you for your insightful comments which have strengthened our manuscript. |
| **Reviewer #2:** | |
| Title |  |
| 1. Does the title give clear idea about the article? No   I think it is better as “Is inadequate preparedness for, response to COVID-19 is associated with stress and burnout among healthcare workers in Ghana?” | Thank you for the suggestion. Given that titles in the form of a question are generally not advised for scientific papers that report findings, we would prefer not to frame the title in the form of a question. We chose our title based on the suggestion to use titles that are concise statements of study findings to draw the attention of readers. However, we defer to the Journal editors on whether the suggested title would be preferred for the manuscript. |
| Also, please write full words in the short title. (HCW) | We have done this. |
| Abstract |  |
| 2. Does the abstract that concisely describes the content and scope of the project and identifies the project’s objective, its methodology and its findings, conclusions, or intended results? Yes | Thank you |
| Introduction |  |
| 3. Does the introduction give clear idea about the article? YES | Thank you |
| Please write references in same fashions. Example, (line 3rd of introduction, ----people as of August 10th, 2020 (1). And line 5th of introduction ……World Health Organization (WHO)(2). | We have checked the in-text references and they are consistent. The mention of “WHO” is not a reference, but rather an abbreviation for World Health Organization. We have restructured the sentence to make this clear. |
| Make Times New Roman style “perceived preparedness” | We have checked to confirm that all formatting is in Times New Roman style font. |
| Methods |  |
| 4. Did methodology part is clear? NO  -I haven’t seen the importance of narrating “2.1. Context…….” under methods. | We thought it would be appropriate to describe the context for people who might not be familiar with it to situate the study. We think this is important, but can delete it if you feel strongly about not describing the context. |
|  |  |
| -You were collecting the data through social media. Your sampling seem non probability because participants those only your friends. | We acknowledge that this was a non-probability sample as described in our method. Probability sampling was not feasible given the situation at the time of the study due to the COVID-19 pandemic, but we thought it was important to conduct this study to provide data to inform policy discussions on the country’s COVID-19 response.  It is however inaccurate to say participants are only our friends. As noted in the methods, we disseminated survey links to Facebook and WhatsApp pages of different professional groups, graduation year groups, and regional groups of HCWs, as well as to leaders of professional organizations and Ghana Health Service directors to share with members of their groups. This approach was used to ensure that we reached a diverse sample of health workers outside of our networks. The sampling method is described in our Methods section, and we discuss the limitations of a non-probability sample in our Discussion section on page 18. |
| - I am afraid of the quality of data. | Per other subsequent comments, this concern appears to be related to recoding of variables. We recoded some variables to avoid very small categories for the regression analysis. We did not think it was important to go into these details. But given your concern, we have added notes to the tables, highlighting composition of recoded categories. |
| -Well, if sociodemographic of participants included | Sociodemographic characteristics of respondents are shown on table 1. We have modified the table title to reflect this. |
| 5. It is well if sub-headed methodology part as: study setting and period, study design, and so on. | We have added the suggested heading(s). |
| 6. Did you conduct a pretest? If yes, how and where? If no, why? | Yes, we pretested the survey with HCWs in Ghana before the actual study. We have added this to the methods on page 8. |
| 7. What language/s used for data collection? | English: We have added this |
| 8. You have no inclusion and exclusion criteria. Please give more clarification for this. | The inclusion criteria were identifying as a HCW based in Ghana, which by default meant exclusion criteria was not identifying as a HCW, and not based in Ghana. We have noted the eligibility criteria in the methods to clarify this on page 8. |
| 9. What do you think about voluntary participation in your study? | As noted in our limitations, voluntary participation is a limitation as volunteers may respond differently from non-volunteers. But as mandated by ethical guidelines from the Institutional Review Board, participation in any study should be voluntary and so this does not invalidate the findings. |
| Results |  |
| 10. You categorized professional type to Doctor, Nurse related and Other. First, what are others? Please specify. Second, it is not recommended to use for more than 5% for others. But yours is 17.6% | We acknowledge this concern. As noted in text, the other professionals, included medical laboratory professionals, disease control officers, nutritionists and other allied health care workers. We have also added this as footnotes in the tables. We grouped these together, because the number of people in these sub-groups was small compared to doctors and nurses and separating them out resulted in very small categories. |
| 11. How you classify years of experiences and age of participants? | The classification was based both on the distribution of the data and to obtain conceptually meaningful categories. We however note in the text that “The average age of respondents was 34.2 years (SD=6.0), with 8.2 years of professional experience (SD=5.6).” |
| 12. You classify marital status in to married and single. What others? (This is what I said quality of data) | There were too few respondents in the other groups, so these were recoded into the two categories. The married category includes 10 people (2%) who were previously married (widowed, separated, or divorced). We have added this as footnotes in the table. |
| 13. Revise the references as the journal guideline | The Journal’s reference guideline is Vancouver, which we used to format our references. We have reviewed the references and verified that they meet the Vancouver guidelines. |
| 14. Tables titles are not written in the standard form. | We have edited the Table titles to fit a more standard form. |
|  | Thank you for your insightful comments to strengthen the paper. |
