# Supplementary material for: Inadequate preparedness for response to COVID-19 is associated with stress and burnout among healthcare workers in Ghana
Source: PLoS One. 2021 Apr 16;16(4):e0250294. doi: 10.1371/journal.pone.0250294 (PMC8051822; doi:10.1371/journal.pone.0250294)
Supplement: Attachment — Submitted filename: Second revision_response to reviewers.docx [file pone.0250294.s008.docx]

Dear Dr. Lionetti,

Thank you for the opportunity to revise and resubmit our manuscript. We have given consideration to the citation suggestion you made and have revised our manuscript accordingly. Below is a clarification and explanation of the revision made in response to the review.

Sincerely,

Manuscript authors

**Response to comments**

Additional Editor Comments (if provided):

The authors have improved the manuscript, but in light of revised version it is important that authors highlight potential comorbidities that affect the enrolled patients (i.e.: cardiovascular disease, obesity). Indeed, recent preclinical study demonstrated that psychosocial stress exerts bigger detrimental effect in the presence of overweight/obesity. Moreover, cardiac function is also impaired following exposure to psychosocial stress since significant oxidative stress occurs in both brain and heart (please see EBioMedicine. 2019 Sep;47:384-401). Since COVID19 induce severe inflammatory response and impair cardiovascular function, authors should discuss their data in light of abovementioned study.

**Response:** We acknowledge this concern. But our study did not enroll patients. Rather it enrolled healthcare workers to examine how their preparedness to respond to COVID-19 is associated with their stress and burnout using psychosocial measures. We have carefully reviewed the paper referenced (EBioMedicine. 2019 Sep;47:384-401) and it does not appear directly relevant to our paper given its focus on biological mechanisms and effects of psychosocial stress on obese mice. Thus, citing the suggested paper would appear out of context in our paper, which focuses on how preparedness level of healthcare workers is associated with psychosocial measures of burnout and stress, not the effects of stress and burnout. We have, however, acknowledged in the limitations section that exploring the effects of stress and burnout experienced by healthcare workers and the associated biological mechanisms was beyond the scope of our paper and that future research may be needed to examine these mechanisms.

**Review 1 Comments to the Author**

1. If the authors have adequately addressed your comments raised in a previous round of review and you feel that this manuscript is now acceptable for publication, you may indicate that here to bypass the “Comments to the Author” section, enter your conflict of interest statement in the “Confidential to Editor” section, and submit your "Accept" recommendation.

Reviewer #1: All comments have been addressed

**Response:** Thank you

2. Is the manuscript technically sound, and do the data support the conclusions?

The manuscript must describe a technically sound piece of scientific research with data that supports the conclusions. Experiments must have been conducted rigorously, with appropriate controls, replication, and sample sizes. The conclusions must be drawn appropriately based on the data presented.

Reviewer #1: Yes

**Response**: Thank you

3. Has the statistical analysis been performed appropriately and rigorously?

Reviewer #1: Yes

**Response**: Thank you

4. Have the authors made all data underlying the findings in their manuscript fully available?

The [PLOS Data policy](http://www.plosone.org/static/policies.action#sharing) requires authors to make all data underlying the findings described in their manuscript fully available without restriction, with rare exception (please refer to the Data Availability Statement in the manuscript PDF file). The data should be provided as part of the manuscript or its supporting information, or deposited to a public repository. For example, in addition to summary statistics, the data points behind means, medians and variance measures should be available. If there are restrictions on publicly sharing data—e.g. participant privacy or use of data from a third party—those must be specified.

Reviewer #1: Yes

**Response**: Thank you

5. Is the manuscript presented in an intelligible fashion and written in standard English?

PLOS ONE does not copyedit accepted manuscripts, so the language in submitted articles must be clear, correct, and unambiguous. Any typographical or grammatical errors should be corrected at revision, so please note any specific errors here.

Reviewer #1: Yes

**Response**: Thank you

6. Review Comments to the Author

Please use the space provided to explain your answers to the questions above. You may also include additional comments for the author, including concerns about dual publication, research ethics, or publication ethics. (Please upload your review as an attachment if it exceeds 20,000 characters)

Reviewer #1: I was pleased to receive and review for the second time the article entitled "Inadequate preparedness for response to COVID-19 is associated with stress and burnout among healthcare workers in Ghana", regarding psychopathological impact of the pandemic on the most affected category in terms of mental well-being.
The authors have exhaustively clarified my doubts, responding privately to my comments or adding specific requests in the text. In particular: information regarding sampling methods, exclusion / inclusion criteria for participants, composition of questionnaires, number and characteristics of drop outs, "overlapping" of the samples for stress and burnout, relationship with the previous paper, references from news sites .

I believe that the information added corresponds to my requests and is sufficient to strengthen the manuscript.

**Response:** Thank you.

7. PLOS authors have the option to publish the peer review history of their article ([what does this mean?](https://journals.plos.org/plosone/s/editorial-and-peer-review-process#loc-peer-review-history)). If published, this will include your full peer review and any attached files.


If you choose “no”, your identity will remain anonymous but your review may still be made public.


**Do you want your identity to be public for this peer review?** For information about this choice, including consent withdrawal, please see our [Privacy Policy](https://www.plos.org/privacy-policy).

Reviewer #1: No
